# Supplementary material for: Combined evaluation of Geriatric nutritional risk index and Neutrophil to lymphocyte ratio for predicting all-cause and cardiovascular mortality in hemodialysis patients
Source: PLoS One. 2023 Jun 29;18(6):e0287696. doi: 10.1371/journal.pone.0287696 (PMC10310003; doi:10.1371/journal.pone.0287696)
Supplement: Attachment — Submitted filename: Response to Reviewers.doc [file pone.0287696.s002.doc]

**Response the academic editor：**

1. Please ensure that your manuscript meets PLOS ONE's style requirements, including those for file naming. The PLOS ONE style templates can be found at
   <https://journals.plos.org/plosone/s/file?id=wjVg/PLOSOne_formatting_sample_main_body.pdf> and
   <https://journals.plos.org/plosone/s/file?id=ba62/PLOSOne_formatting_sample_title_authors_affiliations.pdf>

Response：We have revised the manuscript in accordance with the article format required by POLS ONE.

2. Thank you for stating the following financial disclosure:
      "Funding: This research was funded by the Nanjing Administration of Traditional Chinese Medicine, Project name: Nanjing Traditional Chinese Medicine     Youth Talent Training Program,(NO.ZYQ20053).
       YES - Specify the role(s) played."
    
   Please state what role the funders took in the study.  If the funders had no role, please state: "The funders had no role in study design, data collection and analysis, decision to publish, or preparation of the manuscript."
   If this statement is not correct you must amend it as needed.
   Please include this amended Role of Funder statement in your cover letter; we will change the online submission form on your behalf.

Response：Annotations have been added to the manuscript

3. We note that you have indicated that data from this study are available upon request. PLOS only allows data to be available upon request if there are legal or ethical restrictions on sharing data publicly. For more information on unacceptable data access restrictions, please see [http://journals.plos.org/plosone/s/data-availability#loc-unacceptable-data-access-restrictions](http://journals.plos.org/plosone/s/data-availability#loc-unacceptable-data-access-restrictions).

In your revised cover letter, please address the following prompts:

a) If there are ethical or legal restrictions on sharing a de-identified data set, please explain them in detail (e.g., data contain potentially sensitive information, data are owned by a third-party organization, etc.) and who has imposed them (e.g., an ethics committee). Please also provide contact information for a data access committee, ethics committee, or other institutional body to which data requests may be sent.

b) If there are no restrictions, please upload the minimal anonymized data set necessary to replicate your study findings as either Supporting Information files or to a stable, public repository and provide us with the relevant URLs, DOIs, or accession numbers. For a list of acceptable repositories, please see [http://journals.plos.org/plosone/s/data-availability#loc-recommended-repositories](http://journals.plos.org/plosone/s/data-availability#loc-recommended-repositories).

We will update your Data Availability statement on your behalf to reflect the information you provide.

Response：Article data can be used publicly, the original data has been provided to the editorial department when submission, we have changed in the declaration.

4. Your ethics statement should only appear in the Methods section of your manuscript. If your ethics statement is written in any section besides the Methods, please delete it from any other section.

Response：We have deleted the relevant content from the article.

5. Please include captions for your Supporting Information files at the end of your manuscript, and update any in-text citations to match accordingly. Please see our Supporting Information guidelines for more information: <http://journals.plos.org/plosone/s/supporting-information>.

Response：We have supplemented the original data used in the article and have no other supplementary information.

**Response Reviewer #1：**

①Introduction; I advise authors to make it clear why they studied NLR in hemodialysis patients as a risk factor for CV mortality. Chronic kidney disease is associated with low grade inflammation (). On the other hand, NLR is associated with various inflammatory conditions such as pancreatitis (Iran J Colorectal Res 2022;10(3):75-86.), diabetes mellitus type 2 (African health sciences, 19(1), 1602-1606), autoimmune disorders (Revista da Associação Médica Brasileira, 63, 1065-1068), and thyroid conditions (Ethiop. J. Health Dev. 2021; 35(3):149-153). Inflammation involve in all of these diseases similar to the chronic kidney disease.

Response：Thank you for your suggestion.We have added relevant content in the manuscript.
Materials&Methods; An inclusion criteria was noted as Age≥18. However, mean age was 63 years. Please revise.

Response：Thank you for your suggestion.We have revised it in the manuscript.

**Response Reviewer #2：**

1. the title is not well worded and too long ("All-cause mortality and cardiovascular mortality"?????)

Response：Thank you for your suggestion.We have added relevant content in the manuscript.
2. Abstract: in Introduction add: "Aim of the study was To investigate the predictive value of the.........."

Response：Thank you for your suggestion.We have revised it in the manuscript.
3. Abstract: methodology - this sentence do not belong here: "COX regression analysis showed that GNRI and NLR were risk factors for death in MHD patient" same one is in the Results section too.

Response：Thank you for your suggestion.We have revised it in the manuscript.
4. Improve English

Response：Thank you for your suggestion.We have revised it in the manuscript.
