# Supplementary material for: Combined evaluation of Geriatric nutritional risk index and Neutrophil to lymphocyte ratio for predicting all-cause and cardiovascular mortality in hemodialysis patients
Source: PLoS One. 2023 Jun 29;18(6):e0287696. doi: 10.1371/journal.pone.0287696 (PMC10310003; doi:10.1371/journal.pone.0287696)
Supplement: Attachment — Submitted filename: Response to Reviewers.doc [file pone.0287696.s003.doc]

**Response the academic editor：**

Please review your reference list to ensure that it is complete and correct. If you have cited papers that have been retracted, please include the rationale for doing so in the manuscript text, or remove these references and replace them with relevant current references. Any changes to the reference list should be mentioned in the rebuttal letter that accompanies your revised manuscript. If you need to cite a retracted article, indicate the article’s retracted status in the References list and also include a citation and full reference for the retraction notice.

1. Response：The cited references have been checked and revised.The duplicate references are deleted, the references added in the paper are reordered and the reference codes are reordered

**Response Reviewer #1：**

1.You have mentioned a very good topic in your article but background must be improved. NLR is a novel marker of inflammation derived from routine blood count test. Its association with inflammation has been reported in inflammatory bowel disease (Wiener klinische Wochenschrift (2015)127:262-265. DOI 10.1007/s00508-014-0683-5), diabetes mellitus (Afri Health Sci. 2019;19(1):1602-1606. DOI: 10.4314/ahs.v19i1.35), and gastrointestinal conditions (Iran J Colorectal Res. 2022;10(3):75-86. doi: 10.30476/ACRR.2022.97244.1160). Improve the background please.
Best Regards...

Response：Thank you for your suggestion.We have added relevant content in the manuscript.

**Response Reviewer #2：**

1.In Results section, for number of patients (50 and 29) except total number write values in percentages (%)

Response：Thank you for your suggestion.We have revised it in the manuscript.

2. Three last sentences in Introduction should be written on better way and they should have better formulation in context of Introduction ("No studies have used GNRI and NLR combinations to predict prognosis in MHD patients. GNRI and NLR are clinically relevant indicators of mortality in MHD patients and contribute to early diagnosis and intervention. The study was based on data from two hemodialysis centers, focusing on the impact of GNRI and NLR on all-cause mortality in MHD patients."

Response：Thank you for your suggestion.We have revised it in the manuscript.

3.  "The study was based on data from two hemodialysis centers" - this information should be part of Materials and Methods

Response：Thank you for your suggestion.We have revised it in the manuscript.

4. In Materials and Methods section write the type of the study

Response：Thank you for your suggestion.We have revised it in the manuscript.

5. Inclusion and Exclusion criteria should include more information about patients

Response：Thank you for your suggestion.We have revised it in the manuscript.

6. What about Inform consent of the patients? Did you provide it?

Response：Due to the retrospective nature of the study, informed conssent was waived.The study was approved by the ethic committee of our hospital.

7. In Methods section include formula for NLR calculation

Response：Thank you for your suggestion.We have revised it in the manuscript.

8. In Materials and Methods section include information about all parameters that you was analyzed in the study and explain (for example: hemoglobin, blood phosphorus, platelet-lymphocyte ratio(PLR)...)

Response：Thank you for your suggestion.We have revised it in the manuscript.

9.In Statistical processing section, explain how you determined correlation between two variables (Did you use Pearson or Sperman correlation coefficient?)

Response：Person test was used to analyze the correlation between two variables.Modifications were made in statistical methods.
